# Role of IL-36 Cytokines in the Regulation of Angiogenesis Potential of Trophoblast Cells

**DOI:** 10.3390/ijms22010285

**Published:** 2020-12-30

**Authors:** José M. Murrieta-Coxca, Ruby N. Gutiérrez-Samudio, Heba M. El-Shorafa, Tanja Groten, Sandra Rodríguez-Martínez, Mario E. Cancino-Diaz, Juan C. Cancino-Diaz, Rodolfo R. Favaro, Udo R. Markert, Diana M. Morales-Prieto

**Affiliations:** 1 Placenta Lab, Department of Obstetrics, University Hospital Jena, 07740 Jena, Germany; JoseMartin.MurrietaCoxca@med.uni-jena.de (J.M.M.-C.); Ruby.Gutierrez@med.uni-jena.de (R.N.G.-S.); Heba.Shorafa@med.uni-jena.de (H.M.E.-S.); Tanja.Groten@med.uni-jena.de (T.G.); rodolfo.favaro@med.uni-jena.de (R.R.F.); 2 Departamento de Inmunología y Microbiología, Instituto Politécnico Nacional, Escuela Nacional de Ciencias Biológicas, Mexico City 11340, Mexico; sandrarodm@yahoo.com.mx (S.R.-M.); mecancinod@gmail.com (M.E.C.-D.); jccancinodiaz@hotmail.com (J.C.C.-D.)

**Keywords:** pregnancy, trophoblast, IL-36 cytokines, migration, angiogenesis, microRNAs

## Abstract

IL-36 cytokines (the agonists IL-36α, IL-36β, IL-36γ, and the antagonist IL-36Ra) are expressed in the mouse uterus and associated with maternal immune response during pregnancy. Here, we characterize the expression of IL-36 members in human primary trophoblast cells (PTC) and trophoblastic cell lines (HTR-8/SVneo and JEG-3) and upon treatment with bacterial and viral components. Effects of recombinant IL-36 on the migration capacity of trophoblastic cells, their ability to interact with endothelial cells and the induction of angiogenic factors and miRNAs (angiomiRNAs) were examined. Constitutive protein expression of IL-36 (α, β, and γ) and their receptor (IL-36R) was found in all cell types. In PTC, transcripts for all IL-36 subtypes were found, whereas in trophoblastic cell lines only for *IL36G* and *IL36RN*. A synthetic analog of double-stranded RNA (poly I:C) and lipopolysaccharide (LPS) induced the expression of IL-36 members in a cell-specific and time-dependent manner. In HTR-8/SVneo cells, IL-36 cytokines increased cell migration and their capacity to interact with endothelial cells. *VEGFA* and *PGF* mRNA and protein, as well as the angiomiRNAs miR-146a-3p and miR-141-5p were upregulated as IL-36 response in PTC and HTR-8/SVneo cells. In conclusion, IL-36 cytokines are modulated by microbial components and regulate trophoblast migration and interaction with endothelial cells. Therefore, a fundamental role of these cytokines in the placentation process and in response to infections may be expected.

## 1. Introduction

The establishment of pregnancy requires a finely balanced interaction between embryonal and maternal tissues that is regulated by hormones, cytokines, and other regulatory systems. Several studies in mammals have shown that inflammation is crucial at the maternal-fetal interface, both at implantation and parturition [1,2]. Human placentation is characterized by extravillous trophoblast migration, invasion into the decidua, and replacement of endothelial cells in spiral arteries. This process allows spiral artery remodeling to increase blood supply to the developing fetus [3]. Abnormal inflammation is associated with trophoblast failure to migrate and invade into blood vessels and to acquire the endothelium-like phenotype. This failure contributes to pregnancy pathologies such as preeclampsia, intrauterine growth restriction, and other pregnancy associated pathologies (reviewed in [4]).

The IL-36 group of cytokines comprises three pro-inflammatory agonists (IL-36α, IL-36β, and IL-36γ), and one antagonist (IL-36Ra), which are recently getting attention because of their role in inflammatory diseases like psoriasis [5,6,7,8]. All members of the IL-36 family use the same receptor (IL-36R), a heterodimer composed of interleukin-1 receptor-like 2 (IL1RL2) as the ligand binding moiety and the IL-1 receptor accessory protein (IL1RAcP). This receptor induces inflammatory responses through MyD88, MAPK, NF-kB, and AP-1 pathways. IL-36Ra acts as a natural antagonist, whose binding to IL-36R does not trigger IL-1RAcP recruiting, counterbalancing the inflammatory process [9,10].

The role of the IL-36 family in pregnancy remains largely unknown. In mice, high expression of IL-36 was reported in the uterus at estrus stage and during labor, and is highly induced by *Listeria monocytogenes,* demonstrating its role in inflammation and maternal immune response to pathogens [11,12]. In humans, a recent report demonstrated enhanced levels of IL-36Ra in plasma samples of pregnant compared to non-pregnant women, and decreased IL-36Ra levels in extracellular vesicles from preeclampsia versus healthy placentas [13]. So far, these results suggest that the IL-36 system may have an important role for uterine biology and for the immunological processes required in human pregnancy. We have hypothesized that the IL-36 system regulates trophoblast functions, as specifically the communication with endothelial cells, which may be altered by pathogens and in pregnancy complications such as preeclampsia. Therefore, the aim of this work is to elucidate the potential role of IL-36 agonists in trophoblast cells and to identify their association with angiogenic factors. The expected results will contribute to a better understanding of the function of these pro-inflammatory cytokines during pregnancy including their implication in the trophoblast response to microbial components.

## 2. Results

### 2.1. Primary Trophoblast Cells but Not Their Cell Line Counterparts Express all Members of the IL-36 Family

**Figure 1:**
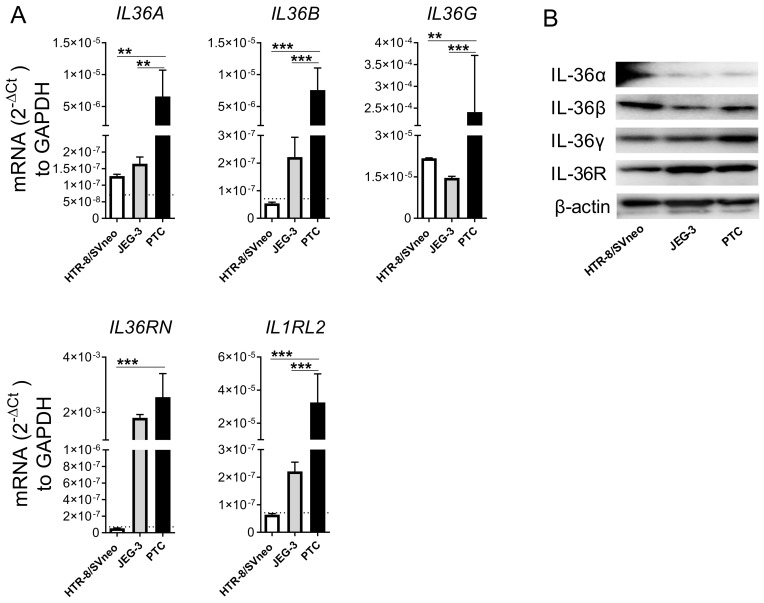
Expression level of IL-36 family members in trophoblast cell models. Trophoblastic cell lines HTR-8/SVneo and JEG-3, and primary trophoblast cells (PTC) from normal placentas were cultured under standard conditions. (**A**) The mRNA levels for IL-36 transcripts were determined by quantitative real-time PCR and normalized using the 2^−ΔCt^ method against Glyceraldehyde 3-phosphate dehydrogenase (GAPDH). PCR results from three independent experiments are shown as mean ± SEM. The horizontal dotted lines denote detection limit (Ct > 40; if not visible it overlaps with the x-axis). One-way ANOVA with Bonferroni’s multiple comparison test. ** *p* < 0.01, *** *p* < 0.001. (**B**) IL-36 (α, β, γ), IL-36R, and β-actin protein detected by Western blotting using 20 μg of respective cell lysate.

The basal mRNA expression of IL-36 family members was examined by qPCR in PTC isolated from third trimester healthy placentas and two widely used trophoblast cell models (HTR-8/SVneo and JEG-3 cells). PTC expressed the transcripts of all four members of the IL-36 family, *IL36G* > 76-fold higher than the other agonists *IL36A* and *IL36B*. In addition, PTC also showed high levels of *IL36RN* (the transcript for IL-36Ra) and *IL1RL2* (the transcript for IL-36R) (Figure 1A). A different expression pattern was found for IL-36 family subtypes in the trophoblastic cell lines. Among the agonists, both cell lines showed similar levels of *IL36G* transcript, which were at least 10-fold lower than those in PTC. *IL36RN* expression was similar in JEG-3 cells and PTC, but was not detectable in HTR-8/SVneo cells. Likewise, in all cell lines transcripts for *IL36A*, *IL36B,* and *IL1RL2* were poorly detected at our detection limit (Ct = 40; Figure 1A). However, we found the protein for IL-36 agonists (α, β, and γ) constitutively expressed in all cell types tested. IL-36α protein was less expressed in comparison with IL-36β and IL-36γ among all tested cell types. Furthermore, all cell types showed great IL-36R protein expression (Figure 1B). These results suggest that HTR-8/SVneo and JEG-3 cell lines as well as PTC are a source of IL-36 cytokines and may be able to respond to IL-36 (α, β, and γ) in an autocrine or paracrine manner.

### 2.2. IL-36 Expression Induced by Poly I:C and LPS Stimulation in Trophoblast Cells

**Figure 2:**
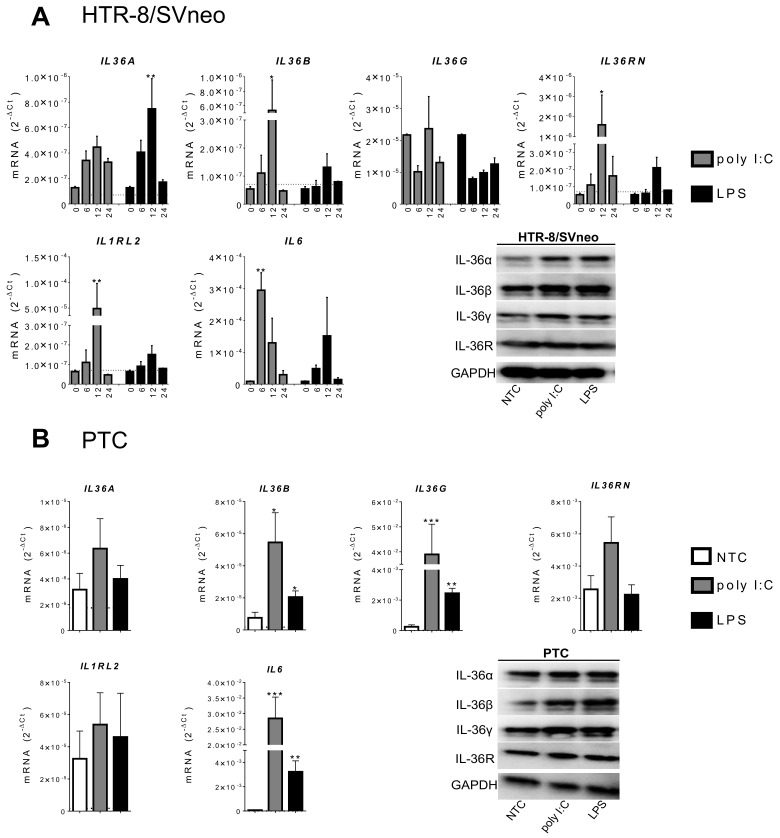
IL-36 expression induced by poly I:C and LPS in HTR-8/SVneo cells and primary trophoblast cells (PTC). (**A**) HTR-8/SVneo cells were stimulated with 25 μg/mL of poly I:C or 100 ng/mL of LPS for 6, 12, and 24 h. (**B**) PTC were stimulated under the same conditions for 12 h. mRNA levels were determined by qPCR and normalized using the 2^−ΔCt^ method to GAPDH. The horizontal dotted lines denote detection limit (Ct > 40; if not visible it overlaps with the x-axis). Results from three independent experiments are shown as mean ± SEM. One-way ANOVA with Dunnett’s multiple comparison test. * p < 0.05, ** *p* < 0.01, *** *p* < 0.001 against the respective 0 h or NTC control. Additionally, in (**A**,**B**), bands show representative Western blotting for detection of IL-36 (α, β, γ), IL-36R, and GAPDH by using 20 μg cell lysate from HTR-8/SVneo and PTC stimulated for 12h. NTC: Non-treated cells.

The presence of bacterial and viral infections impairs trophoblast invasion and disrupts endothelial tubulogenesis ([14,15] and reviewed in [16]). Furthermore, our previous report showed enhanced expression of IL-36 members in murine uterine tissue infected by *Listeria monocytogenes* [11,12,17]. To assess the association of microbial components and IL-36 cytokines in trophoblast cells, the effect of LPS and poly I:C stimulation was investigated. Measuring *IL6* mRNA as common inflammatory cell marker we found a time-dependent response with 25 μg/mL of poly I:C and 100 ng/mL of LPS in HTR-8/SVneo (after 6 h; 33.1- vs. 5.5-fold; Figure 2) and JEG-3 cells (after 6 h; 4.59- vs. 6.86-fold; Supplementary Figure S1). Using the same settings, expression levels of IL-36 subtypes were analyzed. In HTR-8/SVneo cells, a time-dependent response on poly I:C and LPS was observed for *IL36A*, *IL36B*, *IL36RN*, and *IL1RL2* with an expression peak at 12 h (Figure 2A). The high basal expression of *IL36G* mRNA in HTR-8/SVneo shown in Figure 1 was not significantly modulated by poly I:C or LPS (Figure 2). After 12 h, the response to poly I:C was higher than that to LPS in regard to the expression of *IL36B* (9.9- vs. 2.4-fold), *IL36RN* (29.5- vs. 3.1-fold), *IL1RL2* (766.4- vs. 2.4-fold), and *IL6*. LPS was more effective in inducing *IL36A* than poly I:C (5.8- vs. 3.5-fold, respectively). In JEG-3 cells, LPS treatment did not modify the expression of any IL-36 member. A significant induction of *IL36A* (at 6 h) and *IL36G* (at 12 h) mRNA was observed upon poly I:C administration. *IL6* was time-dependently induced in JEG-3 cells by both poly I:C and LPS (Supplementary Figure S1).

These analyses were then repeated in PTC treated for 12 h with the same concentrations of poly I:C and LPS. Although poly I:C induced mRNA expression of all tested IL-36 family members, an increase in expression was only significant for *IL36B* (8.3-fold) and *IL36G* mRNA (315-fold) (Figure 2B). Similarly, LPS induced *IL36B* (3.4-fold) and *IL36G* (15.3-fold). Furthermore, after 12 h poly I:C and LPS stimulation, PTC showed a strong increase of *IL6* mRNA expression (532.8 vs.53.8 fold, respectively).

Intracellular protein expression of IL-36 (α, β, and γ) and IL-36R was further assessed in all three cell types by Western blotting 12 h under stimulation with LPS and poly I:C. IL-36α and IL-36β protein expression was induced in HTR-8/SVneo cells and in PTC after both LPS and poly I:C treatment. A slight increase of IL-36γ was observed upon both stimuli in HTR-8/SVneo cells and was more evident in PTC. No significant changes in IL-36R protein expression were detected in any trophoblast model (Supplementary Figure S1A) and no IL-36 member has changed in JEG-3 cells under the same stimulations (Supplementary Figure S1B). Altogether, these results demonstrate that microbial components trigger IL-36 expression in trophoblast cells, which consequently may cause changes in their function.

### 2.3. IL-36 (α, β, γ) Promote Migration of Trophoblastic Cells

**Figure 3:**
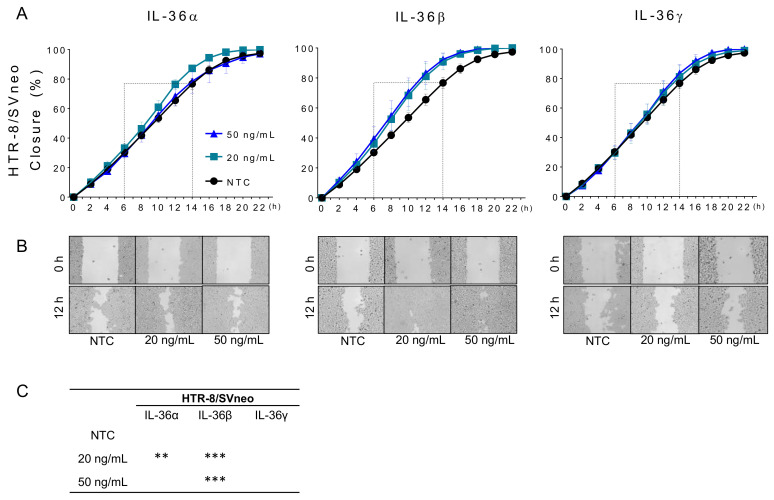
Effect of recombinant IL-36 (α, β, and γ) on trophoblast cell migration. (**A**) Closure rate curves for HTR-8/SVneo cells treated with different concentrations of IL-36α, IL-36β, or IL-36γ. Dashed boxes denote the region-of-interest (ROI) selected for statistical analysis. (**B**) Representative images showing cell distribution at 0 and 12 h. (**C**) Statistical analysis of “wound healing” increase induced by IL-36 (α, β, and γ) compared to non-treated cells (NTC) in the ROI. Results from three independent experiments are shown as mean ± SEM. Two-way ANOVA with Tukey´s multiple comparison test. ** *p* < 0.01, *** *p* < 0.001 against NTC control.

The effect of recombinant IL-36 (α, β, and γ) on the collective migration ability of trophoblastic cells was investigated through applying a “wound healing assay” (Figure 3A,B). A significant increase of HTR-8/SVneo cell migration was observed under treatment with 20 ng/mL of IL-36α as well as 20 and 50 ng/mL of IL-36β (Figure 3C). Because this induction resulted in a coverage of the initially cell free area of >90% after 14 h, the effects were more evident between 6 and 14 h of culture, which corresponded approximately to 30–70% coverage by non-treated cells (NTC) (Figure 3A dashed boxes). At 12 h, the “wound recovery” was increased from 65.5 ± 3.7% (NTC) to 76.3 ± 3.2% by 20 ng/mL of IL-36α and to 81.0 ± 8.8% and 83.3 ± 7.6% by IL-36β at 20 and 50 ng/mL, respectively (Figure 3B). JEG-3 cells migrate less than HTR-8/SVneo cells and 70% coverage was not reached until 24 h. Exogenous IL-36 (α, β, and γ) increased migration ability but the effect was less evident than that in HTR-8/SVneo cells (Supplementary Figure S2). These results suggest that IL-36 cytokines have a positive effect on trophoblast migration. Therefore, this cell line was not used for further functional assays.

### 2.4. Effect of IL-36 (α, β, γ) in the Interaction between Trophoblast and Endothelial Cells

**Figure 4:**
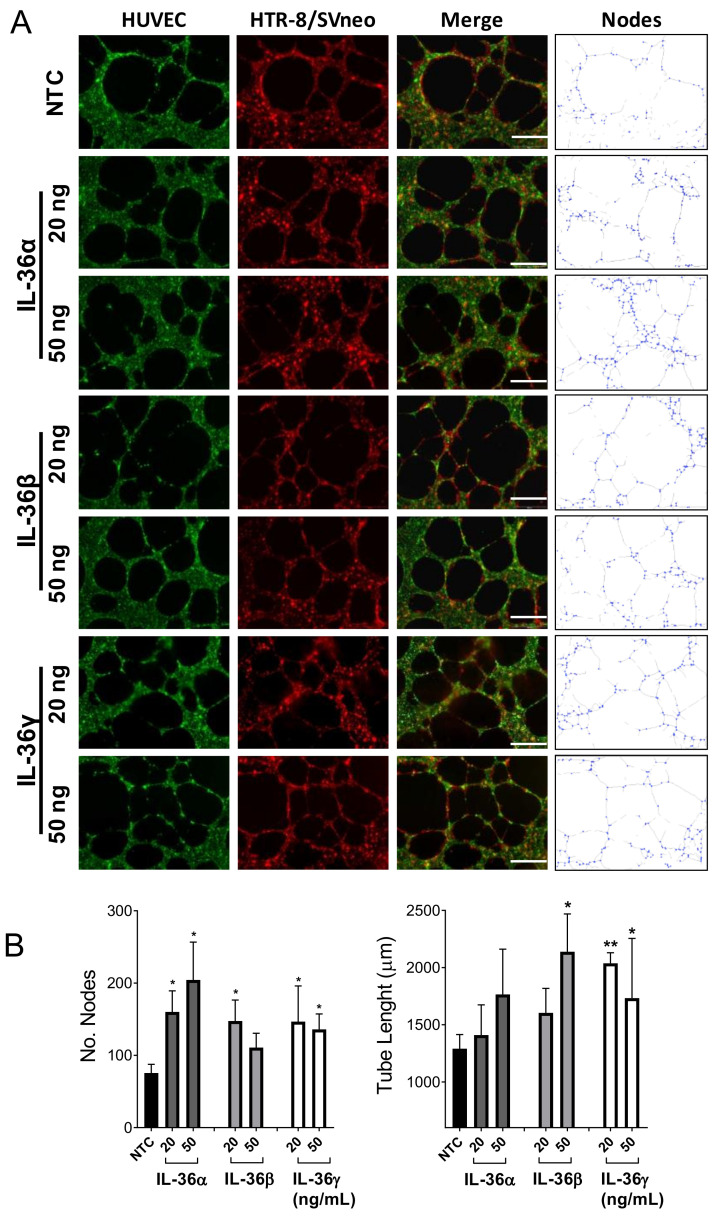
Effects of IL-36 (α, β, and γ) on the interaction between HTR-8/SVneo and endothelial cells. (**A**) Representative images of HUVECs (stained green) and HTR-8/SVneo cells (stained red) after 20 h of co-culture (merge) on a Matrigel^®^-coated μ-plate. Nodes correspond to the analysis of merge images as shown in the right column. (**B**) Bars show the relative number of nodes and the tube length (μm) per condition in comparison with non-treated cells (NTC). Results from five independent experiments are shown as mean ± SEM. One-way ANOVA with Dunnett´s multiple comparison test. * *p* < 0.05, ** *p* < 0.01 for significant differences between respective IL-36 (α, β, and γ) treatments and NTC controls. Scale bar: 50 μm.

To further investigate the effects of IL-36 (α, β, and γ) cytokines on trophoblast function, a 3D model for trophoblast invasion into maternal tissue and spiral artery transformation was utilized [18]. In this model, the endothelial-trophoblast interaction on Matrigel^®^ is determined by the number and stability of nodes within the 3D structure (Figure 4A). In our experiments, HTR-8/SVneo cells (red) were added to previously formed HUVEC structures (green) and allowed to interact for 20 h. During this period, trophoblast cells migrate and invade the HUVEC tubes maintaining and expanding the structure. The number of nodes in the 3D HTR-8/SVneo-HUVEC network increased significantly upon stimulation with IL-36α (20 and 50 ng/mL), IL-36β (20 ng/mL) and IL-36γ (20 and 50 ng/mL; Figure 4B). The positive effect of IL-36 cytokines on the interaction between trophoblast and endothelial cells was further confirmed by assessment of the total tube length (μm) of the structures. Both tested concentrations of IL-36γ and 50 ng/mL IL-36β induced a significant tube elongation after 20 h of stimulation. Also, IL-36α treatment resulted in longer tubes, but not significantly (Figure 4B). Thus, it may be expected that depending on the local concentration, IL-36 (α, β, and γ) affects to different degree the ability of trophoblast cells to migrate and interact with endothelial cells.

### 2.5. IL-36 (α, β, and γ) Induce Expression of Angiogenic Factors in Trophoblast Cells

VEGFA and PGF are strong promoters of angiogenesis and endothelial cell tube formation [14,15], therefore, they play a pivotal role in pregnancy (reviewed in [4]). To further identify the association between IL-36 and the vascular remodeling in pregnancy, expression of *VEGFA* and *PGF* mRNA was investigated in primary and trophoblastic cells upon IL-36 treatment.

**Figure 5:**
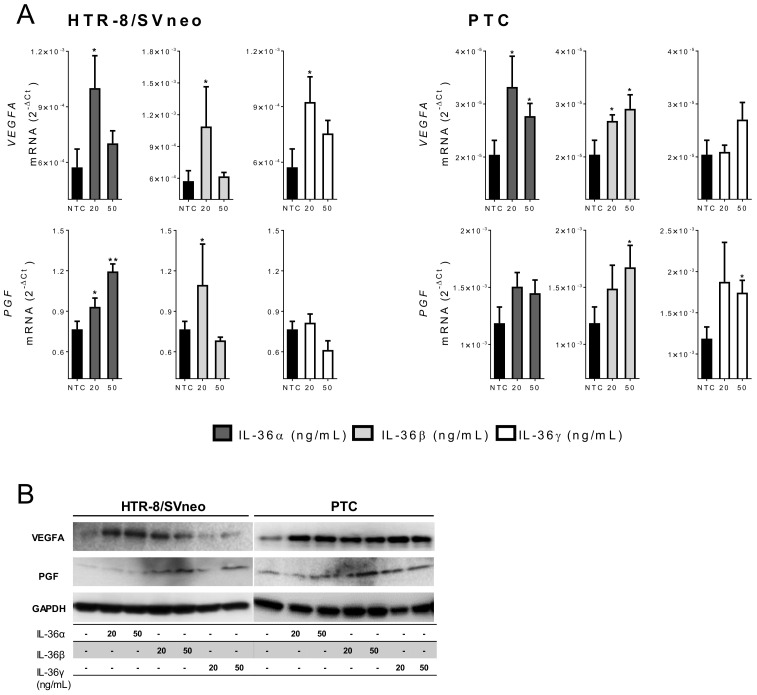
IL-36 (α, β, and γ) induced expression of angiogenic factors. (**A**) HTR-8/SVneo and primary trophoblast cells (PTC) were stimulated with 20 or 50 ng/mL IL-36 (α, β, and γ) for 24 h. The mRNA levels of *VEGFA* and *PGF* were determined by quantitative real-time PCR and normalized to *GAPDH* using the 2^−ΔCt^ method. Results from three independent experiments are shown as mean ± SEM. NTC: Non-treated cells. Unpaired two-tailed Student’s t-test with Welch´s correction. * *p* < 0.05, ** *p* < 0.01 for significant differences to NTC controls. (**B**) VEGFA, PGF, and GAPDH proteins were detected by Western blotting using 20 μg cell lysate from HTR-8/SVneo and PTC stimulated for 24 h.

Basal expression of *VEGFA* and *PGF* was higher in PTC compared to HTR-8/SVneo cells (36.9- and 646-fold, respectively (Supplementary Figure S3). Nevertheless, in both models IL-36 members induced the expression of angiogenic factors. *VEGFA* was significantly increased in HTR-8/SVneo cells by IL-36 (α, β, and γ) cytokines but only at lower concentrations. IL-36β had the strongest effect (3.8-fold) followed by IL-36γ (1.9-fold) and IL-36α (1.7-fold). In PTC, IL-36α and IL-36β had a similar influence inducing *VEGFA* transcription at 20 ng/mL (1.6-fold, and 1.3-fold) and 50 ng/mL (1.3-fold, 1.4-fold), but IL-36γ had no effect (Figure 5A).

*PGF* was upregulated in HTR-8/SVneo cells by IL-36α (dose-dependent) and IL-36β (20 ng/mL), and in PTC by IL-36β and IL-36γ at higher concentrations (1.2- to 2.1-fold; Figure 5A). Finally, we tested the protein expression of VEGFA and PGF under the same stimulation settings. VEGFA was induced by IL-36α and IL-36β in HTR-8/SVneo cells. IL-36 (α, β, or γ) strongly stimulated VEGFA protein expression in PTC (Figure 5B). PGF protein expression was increased upon IL-36β and IL-36γ stimulation in HTR-8/SVneo cells, and upon IL-36 (α, β, or γ) stimulation (at 20 and 50 ng/mL) in PTC (Figure 5B).

Results from the previous section suggested a specific effect of IL-36 cytokines on trophoblastic rather than on endothelial cells. To strengthen these observations, we also investigated expression of *VEGFA* and *PGF* in HUVECs upon IL-36 stimulation. Although HUVECs express IL-36R protein, no significant changes in *VEGFA* or *PGF* mRNA levels were found upon treatment with any IL-36 (α, β, or γ) cytokine (Supplementary Figure S3). These results suggest a different effect of IL-36 cytokines on different cell types: The induction of expression of the investigated angiogenic factors in trophoblast but not in endothelial cells.

### 2.6. microRNA Expression is Regulated by IL-36 Cytokines in Trophoblastic Cells

Previously, we have reported that cytokines present at the implantation site alter the expression of miRNAs in trophoblast cells by affecting pivotal intracellular pathways [19]. Thus, we explored the effect of exogenous IL-36 administration on miRNA expression in trophoblast cells. A set of seven miRNAs was selected comprising angiomiRs identified in other models (miR-132-3p, miR-146a-3p, miR-193b-3p, and miR-378a-5p) and/or reported to be altered in vascular pregnancy pathologies (miR-132-3p, miR-141-3p, miR-141-5p, miR-146a-3p, miR-210-3p).

**Figure 6:**
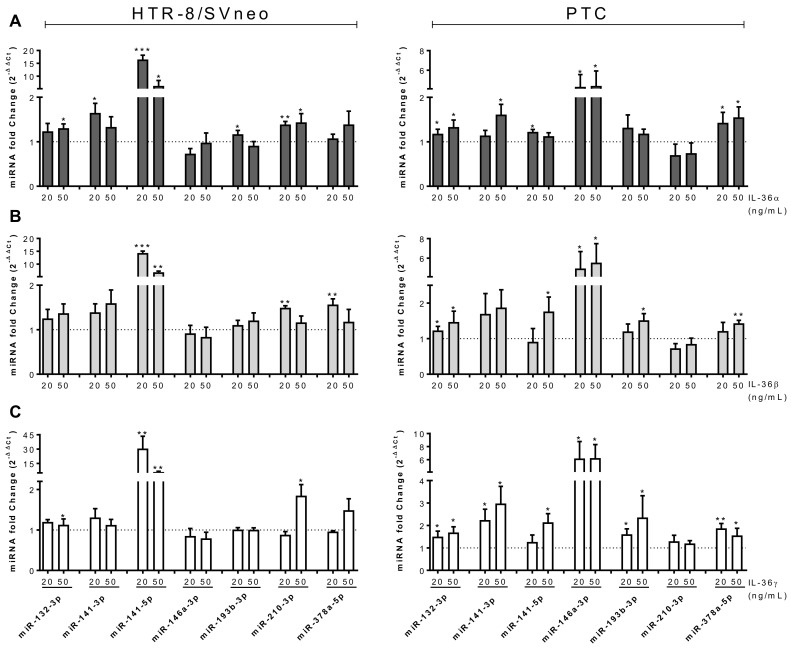
Recombinant IL-36 induces expression of miRNAs. HTR-8/SVneo cells (left) and PTC (right) were stimulated with 20 or 50 ng/mL of (**A**) IL-36α, (**B**) IL-36-β, or (**C**) IL-36γ) for 24 h. miRNA levels were determined by quantitative real-time PCR and normalized using the 2^−ΔΔCt^ method to RNU48 and non-treated cells. Results from three independent experiments are shown as mean ± SEM. Unpaired two-tailed Student’s t-test with Welch´s correction. * *p* < 0.05, ** *p* < 0.01, *** *p* < 0.001 to non-treated cells (dotted line).

In most cases, IL-36 (α, β, and γ) increased miRNA expression, but some differences in the response of PTC and HTR-8/SVneo cells were found. In both models, IL-36α significantly increased the expression of miR-132-3p, miR-141-3p, and miR-141-5p, but additionally increased miR-146a-3p and miR-378a-5p in PTC, as well as miR-193b-3p and miR-210-3p in HTR-8/SVneo cells (Figure 6A).

IL-36β induced miR-141-5p and miR-378a-5p in PTCs and HTR-8/SVneo cells, but also three additional miRNAs in PTC (miR-132-3p, miR-146a-3p, and miR-193b-3p) and miR-210-3p in HTR-8/SVneo cells (Figure 6B).

Finally, IL-36γ administration resulted in common increase of two miRNAs (miR-132-3p, miR-141-5p), four additional miRNAs in PTC (miR-141-3p, miR-146a-3p, miR-193b-3p, and miR-378a-5p) and one in HTR-8/SVneo (miR-210-3p) (Figure 6C).

Among the investigated miRNAs, the greatest effects were found on expression of miR-146-3p in PTC (>5-fold) and miR-141-5p (>10-fold) in HTR-8/SVneo cells.

## 3. Discussion

In the early stages of pregnancy, extravillous trophoblast cells migrate into the maternal decidua, surround the spiral arteries, access their lumen and replace their endothelial cells [3]. This physiological process of vascular remodeling assures proper blood flow to the fetus and, hence, the materno-fetal exchange of nutrients and waste products [14,15]. Disruption of this process results in aberrant placental vascularization and placental pathologies. A complex cytokine network regulates, both positively and negatively, the angiogenic pathways associated with trophoblast-endothelial interaction [20]. Recently, members of the IL-1 superfamily (comprising the IL-36 subfamily) were found in placenta and trophoblast cells, and aberrantly expressed in preeclampsia, a pregnancy disorder often associated with defective trophoblast-driven angiogenesis [13]. However, the functions of the IL-36 subfamily in pregnancy remains largely unknown.

IL-36 cytokines play important roles in innate and adaptive immune responses associated with inflammation in skin, kidneys, joints, brain, and lungs [21]. Several cell types including epithelial (keratinocytes), dendritic, and T helper cells, as well as macrophages and granulocytes are important producers and responders to IL-36, and participate in pro-inflammatory diseases, such as psoriasis [7,22,23]. Here, we report that all members of the IL-36 cytokine family are expressed constitutively in PTC as transcripts and proteins, namely *IL36A* (IL-36α), *IL36B* (IL-36β), *IL36G* (IL-36γ), *IL36RN* (IL-36Ra), and their receptor *IL1RL2* (IL-36R). In our study, the two widely recognized trophoblast cell models JEG-3 and HTR-8/SVneo cells have similar expression of *IL36A* and *IL36G* mRNA and protein in their cell lysates. In HTR-8/SVneo cells, *IL36B* and *IL1RL2* transcripts were below detection limit, but both proteins were detected. It can be argued that in non-treated cells, only low levels of transcripts are available, but under exogenous treatment, mRNA expression can be induced. Differences in the expression of proteins, mRNA, and miRNAs between trophoblastic models and compared to primary cells have been cumulatively reported [24,25,26]. Therefore, two cell models were included in this study to investigate general functions of the IL-36 system in trophoblast biology. JEG-3 cells were derived from choriocarcinoma [27] and are often considered a model for third trimester trophoblast cells and HTR-8/SVneo cells were obtained by immortalization of isolated first trimester extravillous trophoblast cells [28].

Several epidemiological and causal studies have evaluated the relation between maternal infections and pregnancy disorders. In most cases, these studies indicate an association between bacterial and viral infections and the pathogenesis of pregnancy complications [29,30,31]. Previously, we reported a gestational age-dependent expression of IL-36 cytokines in the mouse uterus, which is strongly induced in presence of *L. monocytogenes* infection [11,17]. To examine whether IL-36 cytokines mediate trophoblast response to local infection, we challenged PTC and trophoblastic cells with bacterial (LPS) and viral (poly I:C) components. A transient increase of IL-36 family expression was observed upon both treatments at RNA and protein levels. In our settings, in both PTC and HTR-8/SVneo cells, poly I:C was a stronger inducer of IL-36 cytokine transcripts than LPS with a peak at 12 h of stimulation, whereas, simultaneously, the induction of intracellular protein expression of IL-36 (α, β, and γ) was similar, demonstrating differences in the kinetics of protein and RNA expression. Altogether, these observations agree with other reports highlighting the IL-36 axis function in inflammatory response during bacterial, viral and fungal infections [32,33,34]. They also add to the observation that in keratinocytes low doses of poly I:C already induce expression and release of soluble IL-36γ in a dose- and time-dependent manner [35].

In our study, primary cells were more sensitive to IL-36 stimulation than HTR-8/SVneo cells, and almost no effects were observed in JEG-3 cells. Upon LPS and poly I:C administration, *IL36G* had the highest expression changes in PTC. IL-36γ and IL-36R are also present in epithelial cells of the human female reproductive tract (FRT) and are induced by poly I:C [36]. Pre-treatment with IL-36γ prior to mouse intravaginal viral challenge significantly limited vaginal viral replication and delayed disease onset, decreased disease severity, and increased mice survival [37]. Altogether, these data suggest a role for IL-36γ in host defense against invading pathogens in placental tissues worthy to be further evaluated.

Our results show that IL-36 cytokines are induced by microbial components in trophoblast cells. Treatment with LPS and poly I:C alters trophoblast function which could impair their ability to remodel spiral arteries and contribute to the pathogenesis of pregnancy complications [38,39]. Therefore, we investigated the potential effects of IL-36 on trophoblast behavior. Additional administration of recombinant IL-36γ, which is constitutively expressed by HTR-8/SVneo cells has no effect on their 2D migration. Conversely, both IL-36α and -β promoted migration, but the effect of IL-36α was significant only at low concentrations. This observation is similar to that reported for IL-1β effects on formation of tube-like structures by trophoblast cells. At low concentrations IL-1β increases and at high concentrations decreases their length [40]. Accordingly, we assessed the effect of IL-36 (α, β, and γ) on the capacity of trophoblast to interact with endothelial cells. We found that IL-36 (α, β, and γ) induced a significant increase in the number of nodes and IL-36 (β and γ) additionally favored tube elongation in structures of trophoblastic cells when co-cultured on preformed HUVEC tubes. Node quantification and tube length are accepted parameters for the analysis of in vitro angiogenesis assays, but as they rely, respectively, on the sprouting and growing capacities of the network, which are spatially limited, results may differ among methods [41]. In our results, both parameters pointed out to an enhancing effect of IL-36 cytokines on the interaction of trophoblast and endothelial cells. This goes in line with a report on human endothelial cells co-cultured on a monolayer of primary fibroblasts. Stimulation with IL-36α and IL-36γ results in a significant increase of tubule length and branch point number, which is partly due to the induction of VEGFA expression in fibroblast but not in endothelial cells [42]. However, a more detailed study is needed to elucidate the molecular effects of IL-36 cytokines on trophoblasts and endothelial cells.

Members of the VEGF family, VEGFA and PGF are critical for embryonic angiogenesis [43]. During pregnancy, VEGFA and PGF are expressed in villous and extravillous trophoblast cells, villous vascular endothelium, and decidual natural killer cells, and are altered in pregnancies with adverse outcomes [44,45]. In this study, IL-36 agonists (α, β, and γ) induced *VEGFA* and *PGF* mRNA in a dose- and cell type-dependent manner in trophoblastic cells. In contrast, no changes were observed in *VEGFA* or *PGF* mRNA after IL-36 (α, β, and γ) stimulation in HUVECs (Supplementary Figure S4) although these cells constitutively express IL-36R. Abnormal vascular growth and impaired endothelial function are often associated with pregnancy disorders such as preeclampsia. However, data on *VEGF* and *PGF* expression in normal pregnancy and preeclampsia are still controversial as their mRNA levels have been reported to be decreased, increased or unchanged in preeclamptic placental tissue [45,46,47,48,49,50]. Here, we report induction of VEGFA and PGF mRNA transcript and protein expression in trophoblastic cells by IL-36 and its correlation with promotion of tube formation at low doses.

miRNAs have been established as major regulators of gene expression. Several miRNA species have been suggested to play a role in placental vascular formation by targeting *VEGFA* and other factors (reviewed in [51]). The miRNA profile in trophoblast cells changes with the gestational age and in presence of Leukemia Inhibitory Factor (LIF), a predominant cytokine present in the placenta during early pregnancy [19]. Here, we are reporting a general induction of miRNAs associated with angiogenesis or pregnancy pathologies in trophoblast cells upon IL-36 administration. We identified miR-146a-3p as strongly induced in PTC by IL-36 cytokines. miR-146a was also identified as the most upregulated miRNA in hepatocellular carcinoma (HCC) tissue and cells in vitro. This miRNA enhances endothelial cell activities associated with angiogenesis [52]. Likewise, miR-141-5p and miR-141-3p were induced by IL-36 agonists, in HTR-8/SVneo cells by more than 10-fold. Previously, we have reported upregulation of miR-141-3p in placenta tissue from pregnancies complicated with preeclampsia. This miRNA is exported from trophoblastic cells via extracellular vesicles (EVs) and changes proliferation in target immune cells [53]. In a model of ovarian cancer, miR-141-3p-containing EVs induce the expression of VEGFR-2 in endothelial cells promoting migration and angiogenesis [54]. These observations advocate for a dysregulation of miR-141-3p in malignancies, which can result in aberrant angiogenesis and cell-to-cell communication with immune cells. The implication of the IL-36 axis on these changes in the first stages of pregnancy remains to be elucidated.

Altogether, the results of this study indicate that IL-36 cytokines may play a role in the immune response of trophoblast cells to local infection and/or inflammation. They act as positive regulators of trophoblast migration and angiogenic potential. Their effects seem to be cell type specific and depend on their local concentration.

## 4. Materials and Methods

The Placenta Lab strictly applies quality management and is certified after DIN EN ISO 9001.

### 4.1. Isolation of PTC

PTC were isolated from third trimester placentas as described before [53]. In brief, placental villi were cut into small pieces, washed in sterile PBS with 1% penicillin/streptomycin, and then enzymatically digested at 37 °C in three cycles of 12 min with digestion enzyme solution containing 0.1 mg/mL of DNase type IV, 0.5 mg/mL of Collagenase type IV and 1 mg/mL of Protease type IV (all from Sigma-Aldrich Taufkirchen, Germany) in Dulbecco’s Modified Eagle’s Medium (DMEM) serum free medium (Gibco Thermo Fischer, Carlsbad, CA, USA). Enzymatic activity was stopped by adding equal amount of DMEM supplemented with 10% fetal bovine serum (FBS). Cell suspension was filtered through 100 μm cell strainers, centrifuged for 20 min at 700× *g* and resuspended in supplemented DMEM. A Percoll^TM^ (Merck KGaA, Darmstadt, Germany) gradient (60% and 25%) was used to separate cells. After a spin at 750× *g* for 30 min without brake, the cell layer between 25 and 60% Percoll^TM^ was collected. Trophoblast solution was washed twice with supplemented DMEM medium and centrifuged at 700× *g* for 5 min. Contaminating erythrocytes were eliminated by incubation with 4 mL of 1X RBC lysis buffer (BioLegend, Koblenz, Germany) at room temperature and protected from light for 10 min. After RBC lysis, the cell suspension was centrifuged at 350× *g* for 5 min and resuspended in 2 mL Hanks’ Balanced Salt Solution (HBSS). Leucocytes and fibroblasts were depleted by negative isolation using Dynabeads^®^ (Invitrogen Life Technologies, Darmstadt, Germany) magnetic beads coated with anti-CD45 (BioLegend) and anti-CD82 (Dako Denmark A/S, Glostrup, Denmark) antibodies. The obtained supernatant containing the unbound trophoblast cells was centrifuged at 350× *g* for 5 min. The trophoblast pellet was resuspended in supplemented DMEM and isolated trophoblast cells were seeded in a well plate and cultured at 37 °C in 5% CO_2_ for further experiments.

### 4.2. Cell Culture

JEG-3 and HUVEC cell lines were purchased from the Leibniz Institute—German Collection of Microorganisms and Cell Cultures DSMZ (Braunschweig, Germany). The immortalized cell line HTR-8/SVneo was provided by Dr. Charles H. Graham, (Queen’s University, Kingston, ON, Canada). Cell cultures were performed at 1 × 10^6^ cells in a 75 cm^2^ flask and maintained under standard conditions (37 °C, 5% CO_2_ and humid atmosphere) in DMEM, Ham’s F-12 Nutrient Mix or RPMI 1640 Medium (Gibco Thermo Fischer) for PTC, JEG-3 and HTR-8/SVneo cells, respectively. All media were supplemented with 10% FBS and 1% penicillin-streptomycin antibiotic solution. HUVEC culture was maintained in Endothelial Cell Growth Medium (ECGM) supplemented with 10% FBS and PromoCell Supplement Mix (Promo Cell GmbH, Heidelberg, Germany).

### 4.3. Cell Stimulation

Trophoblastic cell lines (4 × 10^5^ HTR-8/SVneo and JEG-3 per well) were seeded in 6-well culture plates and allowed to attach overnight. Cells were stimulated with 100 ng/mL LPS, 25 μg/mL poly I:C (both from Sigma-Aldrich) or medium alone as control for 6, 12 and 24 h. Based on the results from cell lines, PTC were incubated for 12 h with the same concentrations of LPS and poly I:C.

For the assessment of angiogenic factors and miRNAs, 2 × 10^5^ HTR-8/SVneo, PTC or HUVEC cells were cultured in 12-well plates and then treated for 24 h with 20 or 50 ng/mL of IL-36 (α, β, or γ; ImmunoTools GmbH, Friesoythe, Germany).

After stimulation, cells were harvested for RNA isolation or Western blotting.

### 4.4. RNA Isolation and qPCR

RNA was isolated from cells before and after stimulation using TRIzol reagent (Invitrogen, Darmstadt, Germany). Total RNA concentration was determined in a NanoDrop ND-1000 spectrophotometer (Thermo Fisher Scientific, Wilmington, DE, USA). Samples with A260/A280 ratio >1.8 were stored at −80 °C until being processed. Expression of IL-36 members and IL-6 was determined by reverse transcription using High-Capacity RNA-to-cDNA™ Kit (Applied Biosystems, Foster City, CA, USA). Quantitative real-time PCR was performed using TaqMan assays (*IL36A*, Assay ID: Hs00205367_m1; *IL36B*, Assay ID: Hs00758166_m1; *IL36G*, Assay ID: Hs00219742_m1; *IL36RN*, Assay ID: Hs00202179_m1; *IL1RL2*, Assay ID: Hs00187259_m1; *IL6*, Assay ID: Hs00174131_m1; *VEGFA*, Assay ID: Hs00903127_m1; *PGF*, Assay ID: Hs01119259_m1; and *GAPDH*, Assay ID: Hs02758991_g1) and TaqMan Universal PCR Master Mix reagents (Applied Biosystems). qPCR was run on a Mx3005P qPCR System (Applied Biosystems). Expression of IL-36 members, IL-6, *VEGFA* and *PGF* were normalized using the 2^−ΔCt^ method relative to Glyceraldehyde 3-phosphate dehydrogenase (GAPDH).

Expression level of seven miRNAs (has-miR-132-3p, Assay ID: 000457; has-miR-141-3p, Assay ID: 000463; has-miR-141-5p, Assay ID: 002145; has-miR-146a-3p, assay ID: 000468; has-miR-193b-3p, Assay ID: 002367; has-miR-210-3p, Assay ID: 000512; has-miR-378a-5p, Assay ID: 000567) was tested by applying individual TaqMan miRNA Assays (Applied Biosystems) according to the protocol provided by the supplier. Reverse transcription was performed with miRNA specific stem-loop RT primers and TaqMan MicroRNA Reverse Transcription Kit (Applied Biosystems). Real time PCR was performed using specific TaqMan Assays and TaqMan Universal PCR Master Mix. All reactions were run in duplicates including no-template controls in 96-well plates on Mx3005P qPCR System (Applied Biosystems). Fold changes were calculated by the formula 2^−ΔΔCt^ using RNU48 as housekeeping control and normalized to non-treated cells.

### 4.5. Western Blotting Analysis

HTR-8/SVneo, JEG-3, PTC and HUVEC cell pellets were lysed using RIPA lysis buffer (1% NP-40, 0.1% SDS, 0.5% sodium deoxycholate, 150 mM NaCl and 50 mM Tris–HCl) containing protease inhibitors. Total protein concentrations were assessed using the Pierce™ Micro BCA™ Protein-Assay (Thermo Scientific). Protein extracts were loaded on a 12% precast gel SERVAGel™ (SERVA Electrophoresis GmbH, Heidelberg Germany), and resolved proteins were transferred to a nitrocellulose membrane (Hybond-P; GE Healthcare, Freiburg, Germany). Non-specific binding sites were blocked by incubation with TBS-T containing 5% (*w*/*v*) non-fat dried milk for 1 h at room temperature. Membranes were immunoblotted with specific primary antibodies overnight at 4 °C, followed by 1 h incubation at room temperature with the respective HRP-conjugated secondary antibody. The following primary monoclonal antibodies (goat) were purchased from R&D Systems (Minneapolis, MI, USA) and diluted 1:500, anti-IL-36α (Cat.Nr: AF-1078), anti-IL-36β (Cat.Nr: AF-10998), anti-IL-36γ (Cat.Nr: AF-2320), anti-VEGF (Cat.Nr: AF-293), anti-PGF (Cat.Nr: AF-264). Anti-goat-HRP was diluted 1:5000 (Santa Cruz Biotechnology, Heidelberg, Germany, Cat.Nr: sc-2768) for their detection. Rabbit- anti-IL-36R (Abcam, Cat.Nr: ab180894), -anti-human GAPDH (Cell Signaling, Cat.Nr 2118S) and -anti-β-actin (Cell Signaling, Cat.Nr 4970L) were applied at a 1:500 dilution and detected with anti-rabbit-HRP diluted 1:5000 (Cell Signaling, Cat.Nr 7076P2). To show equal loading, membranes were treated twice with stripping buffer (0.2 M Glycine, 0.1% (*w*/*v*) SDS and 1% Tween20, pH 2.2) for 10 min at room temperature, washed with PBS and TBS-T, and blocked with TBS-T containing 5% (*w*/*v*) non-fat dried milk for 1 h at room temperature before being re-probed with anti-GAPDH (Figure 1) or anti-β-actin (Figure 2) antibodies. Blots were developed using an enhanced chemiluminescence (ECL) detection kit (Millipore, Schwalbach, Germany). Bands were detected by a MF-ChemiBis 3.2 gel documentation system with Totallab TL100 software version 2006 (Biostep GmbH, Jahnsdorf, Germany).

### 4.6. “Wound Healing” Assay (Cell Migration)

For the migration assay, ibidi™ culture inserts (Ibidi GmbH, Cat.Nr. 80209, Gräfelfing, Germany) were used. Briefly, the inserts consist of two chambers separated by a 0.5 mm divider (forming the “wound”), each chamber with a growth area of 0.22 cm^2^. The inserts were set into wells of a 24-well plate by using sterile tweezers and 70,000 cells were transferred to each chamber. After 4 h, cells were adhered, and the culture inserts were gently removed. The wells were filled with 1 mL of medium supplemented with 0, 20 or 50 ng/mL of IL-36 (α, β, or γ) and further incubated for 24 h. The rate of “wound” closure was monitored at regular intervals of 1 h using the JuLI™ Stage automated cell imaging system (NanoEnTek, Seoul, Korea). The JuLI™ Stage software offers image capturing and time-lapse recording with multiposition scanning.

### 4.7. Tube Formation Assay

Wells of an angiogenesis 96 wells μ–plate (“ibiTreat”, Ibidi, Cat.Nr: 89646) were coated with 10 μL growth factor reduced Matrigel^®^ (Corning, Wiesbaden, Germany, Cat.Nr: 356230) (37 °C for 30 min), filled with 50 μL endothelial cell growth medium (ECGM; PromoCell, C-22010) per well and incubated overnight. 6500 HUVEC cells were stained with 10 nM CellTracker™ Green for 30 min at 37 °C (Sigma-Aldrich, Cat.Nr: C2925), added to each well and incubated at 37 °C for at least 4 h to allow tube formation. Medium was removed and co-culture was started by adding 6500 HTR-8/SVneo cells previously stained with CellTracker™ Orange (10 nM, 30 min; Sigma-Aldrich, Cat.Nr: C2927). After 20 h of co-culture, changes in the tube formation were observed and pictures were taken using an Olympus IX-81 inverted system microscope. Tube formation assay was analyzed with the Angiogenesis Analyzer plugin developed by Carpentier, implemented in the software ImageJ. Tube formation and stability were quantified as mean number of unions between three or more tubular structures (nodes) from stimulated and non-stimulated co-cultures. The tube length (μm) was additionally analyzed using the “Tube Formation FastTrack AI Image Analysis” (MetaVi Labs Inc., Bottrop, Germany).

### 4.8. Statistical Analysis

Experiments were repeated independently at least 3 times. Unpaired two-tailed Student’s t-test with Welch´s correction and one-way ANOVA with Bonferroni multiple comparison test were performed for comparisons as indicated in the figure legends using Prism software version 6 (GraphPad, San Diego, CA, USA). A *p* value < 0.05 was considered significant.

## 5. Conclusions

Primary trophoblast cells express basal mRNA levels of IL-36 cytokines. This profile differs from that of trophoblastic cell lines and is altered upon stimulation with microbial components. Our data support a role for IL-36 cytokines in promoting expression of angiogenic factors in trophoblast cells as well as their ability to interact with endothelial cells. Thus, deciphering the biological significance of IL-36 in trophoblast functions constitutes an important issue for further translational and clinical studies.

## Data Availability

The data presented in this study are available on request from the corresponding author.

## Supplementary Materials

Supplementary Materials can be found at https://www.mdpi.com/1422-0067/22/1/285/s1.

## Abbreviations

AECs	Alveolar epithelial cells
DMEM	Dulbecco’s Modified Eagle’s Medium
ECGM	Endothelial Cell Growth Medium
ECM	Extracellular matrix
FRT	Female reproductive tract
GAPDH	Glyceraldehyde 3-phosphate dehydrogenase
HBSS	Hanks’ Balanced Salt Solution
HSV-2	Herpes-simplex-Virus 2
HUVEC	Human umbilical vein endothelial cells
ICAM-1	Intercellular Adhesion Molecule 1
IL-1RAcP	Interleukin-1 receptor accessory protein
IL1RL2	Transcript for IL-36R protein
IL36A	Transcript for IL-36α protein
IL36B	Transcript for IL-36β protein
IL36G	Transcript for IL-36γ protein
IL36RN	Transcript for IL-36Ra protein
LPS	Lipopolysaccharide
MAPK	Mitogen-activated protein kinases
MyD88	Myeloid differentiation primary response 88
NF-kB	Nuclear factor ‘kappa-light-chain-enhancer’ of activated B-cells
NTC	Non-treated cells
PCR	Polymerase chain reaction
PGF	Placental growth factor
PTC	Primary trophoblast cells
RPMI	Roswell Park Memorial Institute medium
VCAM-1	Vascular cell adhesion protein 1
VEGFA	Vascular endothelial growth factor A
