# Supplementary material for: Role of IL-36 Cytokines in the Regulation of Angiogenesis Potential of Trophoblast Cells
Source: Int J Mol Sci. 2020 Dec 30;22(1):285. doi: 10.3390/ijms22010285 (PMC7794747; doi:10.3390/ijms22010285)
Supplement: Supplementary file 1 [file ijms-22-00285-s001.pdf]

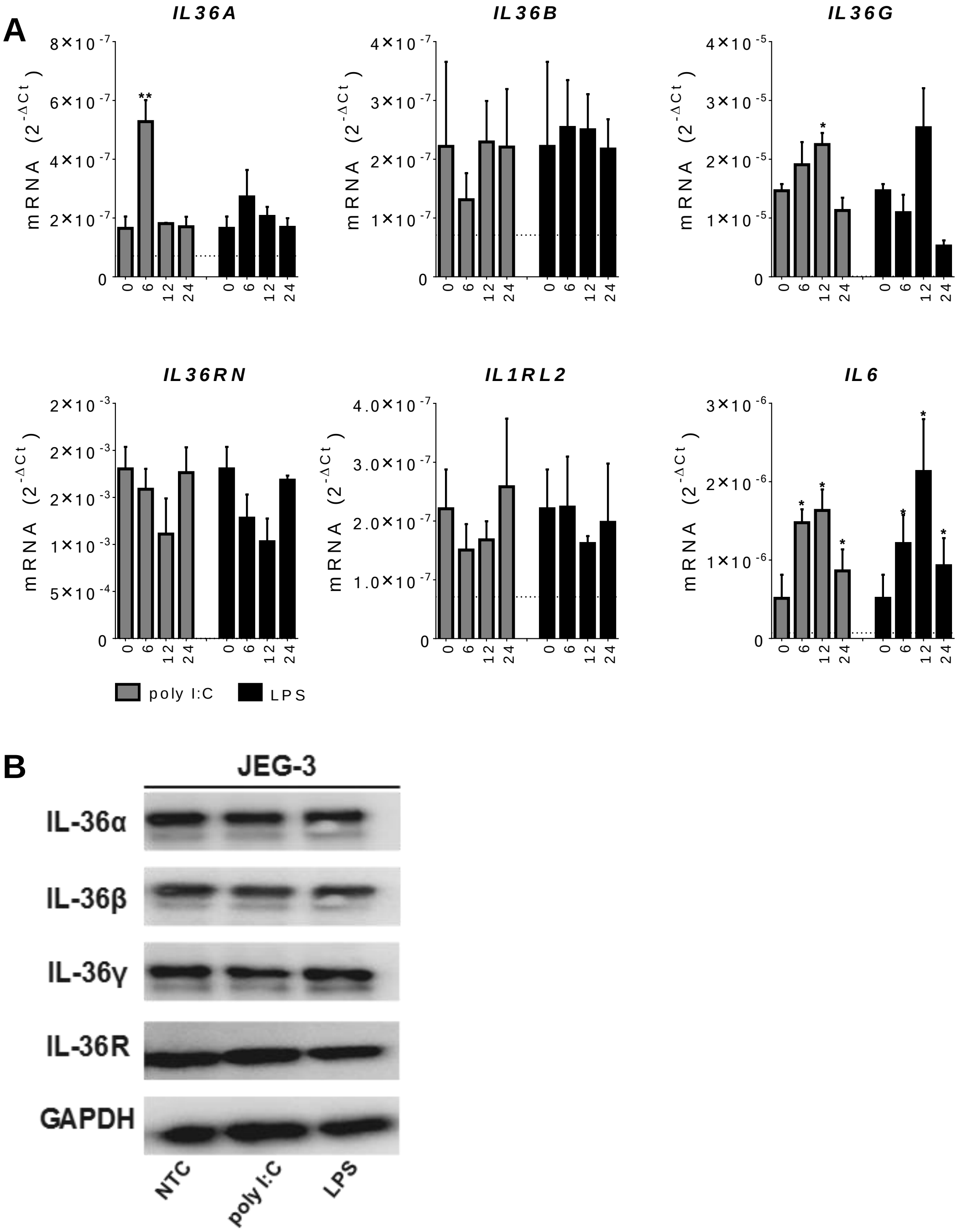

Supplementary Figure S1. IL-36 expression induced in JEG-3 cells after poly I:C and LPS stimulation. JEG-3 cells were cultured under standard conditions. Cells were stimulated with 25 μg/mL of poly I:C, 100 ng/mL of LPS, or medium alone as a control for 6, 12 or 24 h. The mRNA levels of *IL36A*, *IL36B*, *IL36G*, *IL36RN*, *IL1RL2* and *IL6* were determined by quantitative real-time PCR and normalized to *GAPDH* using the $2^{-\Delta Ct}$ method. PCR results are shown as the mean ± SEM (n=3). One-way ANOVA with Dunnett´s multiple comparisons test. *$*p < 0.05$, $**p < 0.01$* for significant differences to 0 h. B) IL-36 (α, β, γ), IL-36R and GAPDH proteins were detected by Western blotting using 20 μg of cell lysate from JEG-3 cells stimulated for 12h with poly I:C or LPS respectively.

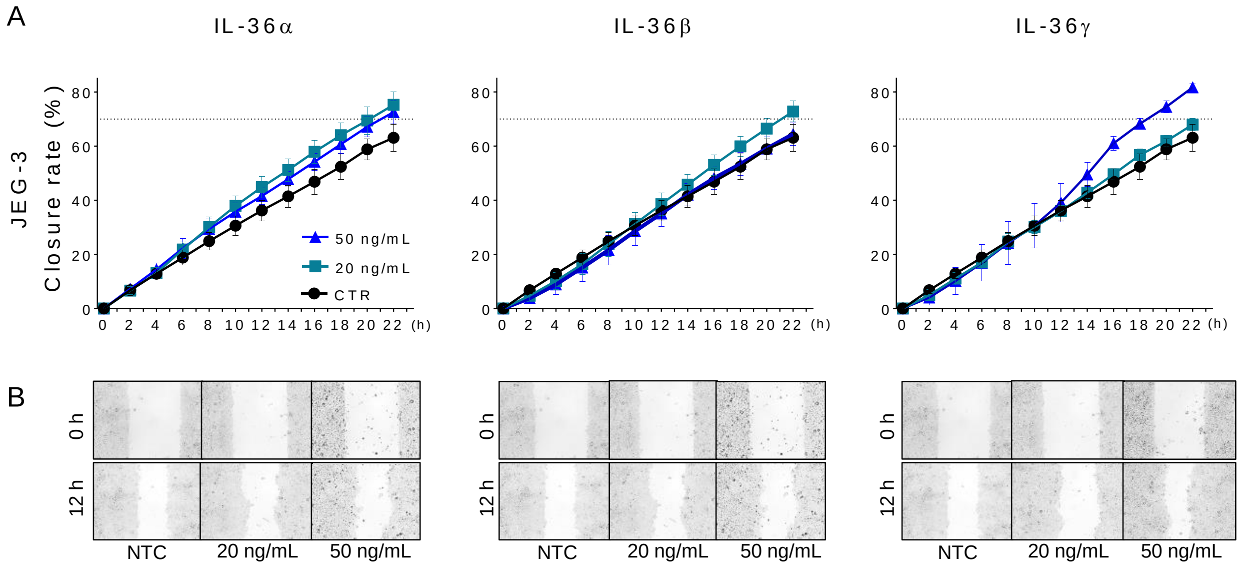

C

	JEG-3		
	IL-36α	IL-36β	IL-36γ
NTC			
20 ng/mL	***		
50 ng/mL	**		**

Supplementary Figure S2. Effect of recombinant IL-36 (α, β and γ) on JEG-3 cell migration. JEG-3 cells were seeded in both chambers of an Ibidi insert separated by a 0.5 mm divider. After 4 h attachment, the insert was removed, and recombinant IL-36 was added. The resultant gap ("wound") was photographed every hour and closure was expressed as percentage of the initial wound. A) Closure rate curves for HTR-8/SVneo cells treated with different concentrations of IL-36α, IL-36β or IL-36γ. Dashed lines denote the 70 % closure rate. B) Representative images showing cell distribution at 0 and 12 h. C) Statistical analysis of "wound healing" increase induced by IL-36 (α, β and γ) compared to non-treated cells (NTC). Results are from three independent experiments and shown as mean ± SEM. Two-way ANOVA with Tukey´s multiple comparisons test. **$p < 0.01$, ***$p < 0.001$ against NTC control.

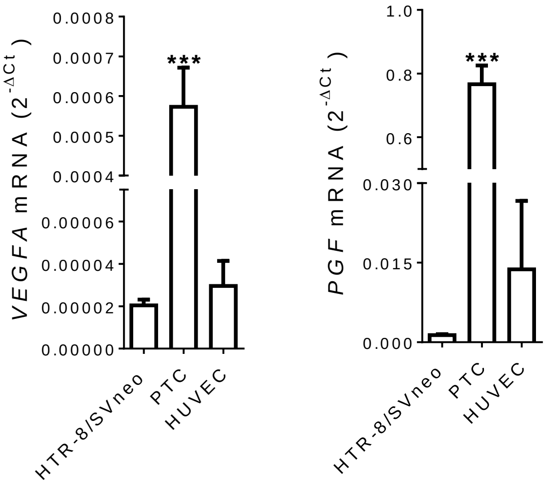

Supplementary Figure S3. *VEGFA* and *PGF* in non-stimulated cells. HTR-8/SVneo, PTC and HUVECs were cultured under standard conditions. The mRNA levels of *VEGFA* and *PGF* were determined by quantitative real-time PCR and normalized to *GAPDH* using the $2^{-\Delta Ct}$ method. PCR results are shown as the mean ± SEM. One-way ANOVA with Dunnett´s multiple comparisons test. *p<0.001* for significant differences between PTC and other cell types.

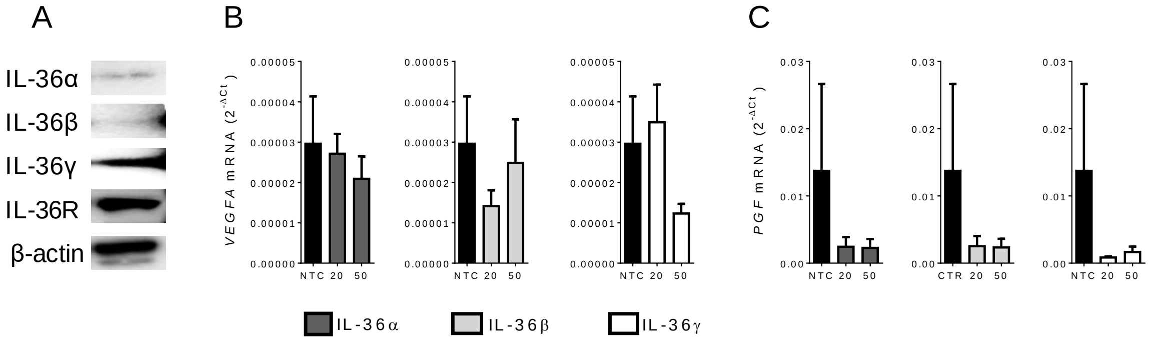

Supplementary Figure S4. HUVECs stimulated with IL-36 (*α*, β and *γ*). A) Western blots showing IL-36 (*α*, β and *γ*) and IL-36R protein expression in non-stimulated HUVECs. Further, HUVECs were cultured under standard conditions and were stimulated with 20 and 50 ng/mL of IL-36 (*α*, β and *γ*) or medium as a control for 24 h. The mRNA levels of **B)** *VEGFA* and **C)** *PGF* were determined by quantitative real-time PCR and normalized to *GAPDH* using the $2^{-\Delta Ct}$ method. PCR results are shown as the mean ± SEM (n=3). Unpaired two-tailed Student's t-test with Welch´s correction.
